# Baseline Renal Function Predicts Hyponatremia in Liver Cirrhosis Patients Treated with Terlipressin for Variceal Bleeding

**DOI:** 10.1155/2017/7610374

**Published:** 2017-09-17

**Authors:** Sung Eun Kim, Dong Min Jung, Ji Won Park, Yeonmi Ju, Bohyun Lee, Hyoung Su Kim, Ki Tae Suk, Myoung Kuk Jang, Sang Hoon Park, Jun Goo Kang, Jae Seung Soh, Hyun Lim, Ho Suk Kang, Sung Hoon Moon, ChulSik Kim, SeongJin Lee, Jong Hyeok Kim, Myung Seok Lee, Dong Joon Kim, Sung-Hee Ihm, ChoongKee Park

**Affiliations:** ^1^Department of Internal Medicine, Hallym University Sacred Heart Hospital, Hallym University College of Medicine, Anyang, Republic of Korea; ^2^Department of Internal Medicine, Hallym University Gangdong Sacred Heart Hospital, Gangdong, Republic of Korea; ^3^Department of Internal Medicine, Hallym University Chuncheon Sacred Heart Hospital, Chuncheon, Republic of Korea; ^4^Department of Internal Medicine, Hallym University Gangnam Sacred Heart Hospital, Seoul, Republic of Korea

## Abstract

**Objectives:**

Terlipressin is safely used for acute variceal bleeding. However, side effects, such as hyponatremia, although very rare, can occur. We investigated the development of hyponatremia in cirrhotic patients who had acute variceal bleeding treated with terlipressin and the identification of the risk factors associated with the development of hyponatremia.

**Design and Methods:**

This retrospective, case-control study investigated 88 cirrhotic patients who developed hyponatremia and 176 controls that did not develop hyponatremia and were matched in terms of age and gender during the same period following terlipressin administration.

**Results:**

The overall change in serum sodium concentration and the mean lowest serum sodium concentration were 3.44 ± 9.55 and 132.44 ± 8.78 mEq/L during treatment, respectively. Multivariate analysis revealed that baseline serum sodium was an independent positive predictor, and the presence of baseline serum creatinine, HBV, DM, creatinine, and shock on admission was independent negative predictors of hyponatremia (*P* < 0.05).

**Conclusion:**

The presence of HBV, DM, the baseline serum sodium, shock on admission, and especially baseline creatinine may be predictive of the development of hyponatremia after terlipressin treatment. Therefore, physicians conduct vigilant monitoring associated with severe hyponatremia when cirrhotic patients with preserved renal function are treated with terlipressin for variceal bleeding.

## 1. Introduction

The mortality from variceal bleeding in the patients with cirrhosis has decreased from approximately 50% to 15–20% due to the development of endoscopic treatments and the early administration of portal pressure-reducing drugs [1, 2]. Variceal bleeding is a lethal complication of cirrhosis that results from portal hypertension. Terlipressin is a synthetic analogue of vasopressin that is effective in controlling acute variceal bleeding [3–6] and is considered to be a safe vasoconstrictor because terlipressin exhibited increased selectivity for the V1 receptor compared with arginine vasopressin. However, several studies have reported patients with variceal bleeding who developed mild to severe hyponatremia upon treatment with standard doses of terlipressin [7–10]. Furthermore, most recent practice guidelines for the management of gastrointestinal bleeding due to portal hypertension recommended that hyponatremia has been occurred in patients under terlipressin therapy, especially in patients with preserved liver function [11]. We also experienced a case of hyponatremic seizure induced by treatment with standard doses of terlipressin in a 52-year-old female who had isolated gastric variceal bleeding secondary to alcoholic liver cirrhosis [12]. Therefore, we thought that the development of hyponatremia may be an important side effect in cirrhotic patients with acute variceal bleeding following treatment with terlipressin. Moreover, there are no reports of the development of hyponatremia after the administration of terlipressin for acute variceal bleeding in patients with hepatocellular carcinomas (HCCs).

Thus, we investigated the development of hyponatremia in cirrhotic patients with and without HCC who had acute variceal bleeding that was treated with the standard doses of terlipressin and attempted to identify the risk factors associated with the development of hyponatremia.

## 2. Materials and Methods

### 2.1. Patients and Study Design

A total of 88 cirrhotic patients with or without HCC who developed hyponatremia following the administration of terlipressin (Ferring International, Saint-Prex, Switzerland) for acute variceal bleeding were retrospectively enrolled from the Hallym University Sacred Heart Hospital, Korea, from January 2007 to March 2012. For the purpose of comparison, 176 cirrhotic patients with or without HCC who had not developed hyponatremia and who matched the patients who developed hyponatremia in age and gender were selected during the same period. The diagnosis of cirrhosis was based on clinical evidence of portal hypertension (the presence of esophagogastric varices and/or ascites plus splenomegaly with a platelet count <100,000/mm^3^) or ultrasonography or computed tomography features suggesting cirrhosis, including liver surface irregularities and nodularities [13]. HCC was diagnosed according to the noninvasive diagnostic criteria of the American Association for the Study of Liver Diseases or histopathological examination [14].

Acute variceal bleeding was defined as a diagnosis made by an endoscopist with no other defined cause of bleeding. Endoscopy was performed when a cirrhotic patient with or without HCC exhibited a clinical suspicion of bleeding, such as hematemesis, melena, hematochezia, or an unexplained hemoglobin decrease of >2 g/dL. Endoscopic treatments, such as endoscopic variceal band ligation and sclerotherapy for variceal bleeding, were performed at the time of diagnosis in most patients. All patients were initially intravenously injected with 2 mg terlipressin every 4 hours and subsequently injected with additional 1 mg doses every 4 hours for a total of 5 days [3–6]. Hyponatremia was defined as a decrease in sodium concentration >5 mmol/L. None of the patients had syndrome for inappropriate antidiuretic hormone, pneumonia, pulmonary tuberculosis, small cell lung cancer, or infections of the central nervous system. The study protocol was reviewed and approved by the institutional review board of Hallym University Sacred Heart (HUSHHIRB/EC2016-I014), and the study was conducted in accordance with the ethical guidelines of the 1975 Declaration of Helsinki.

### 2.2. Clinical and Laboratory Assessment

The baseline serum sodium concentration was taken as that observed on admission, and the lowest level of sodium concentration obtained between 2 and 5 days after terlipressin administration was recorded. All patients were subjected to hepatic and renal function tests, cell blood counts, and daily serum electrolyte tests during the period of terlipressin treatment. Cell blood counts, hepatic and renal function tests, and serum electrolyte tests were performed every 2-3 days until discharge or death following the end of terlipressin administration.

### 2.3. Statistical Analyses

The statistical analyses were performed using SPSS version 18.0 (SPSS Inc., Chicago, IL, USA). All data are expressed as the means ± the SD or the medians with the ranges. The continuous data were analyzed with Student's *t*-tests for equal variance or Mann–Whitney tests for unequal variance, and categorical valuables were investigated with Pearson's *χ*^2^ tests. Univariate and multivariate logistic regression model analyses were utilized to determine the independent predictors of the development of hyponatremia and the main factors associated with in-hospital mortality. *P* < 0.05 was considered to indicate significance.

## 3. Results

### 3.1. Patient Characteristics

The baseline characteristics of all of the patients (*n* = 264) are summarized in Table 1. Among these patients, 88 patients who developed hyponatremia were included as the case group, and 176 patients who were matched for age and sex were randomly selected as the control group using a computer-generated random number table. In the case group, seventy-three (83%) patients were male, and the mean age was 56.3 years. In the control group, one hundred forty-six (83%) patients were male, and the mean age was 57.5 years. All enrolled patients had cirrhosis. The prevalence of hepatitis B surface antigen positivity and diabetes mellitus (DM) was significantly higher in the control group than in the case group (47.2% versus 30.7%; *P* = 0.011; and 47.9% versus 19.3%; *P* = 0.029). The Child-Pugh score and the Model for End-Stage Liver Disease (MELD) score were used to assess decreased liver function, and both were significantly higher in the control group than in the case group (9.5 ± 2.7 versus 8.4 ± 2.3; *P* = 0.001, 20.7 ± 10.2 versus 14.0 ± 5.0; *P* < 0.001). The serum levels of creatinine were significantly higher in the control group compared with the case group (1.6 ± 1.4 mg/dL versus 0.8 ± 0.4 mg/dL; *P* < 0.001). The baseline sodium levels were 137.8 ± 4.5 mmol/L in the case group and 134.1 ± 7.1 mmol/L in the control group (*P* < 0.001). The lowest sodium levels after terlipressin treatment were 125.9 ± 6.8 mmol/L in the case group and 137.2 ± 7.8 mmol/L in the control group (*P* < 0.001). There was statistically insignificant difference between the groups in terms of age, sex, alcohol consumption, hepatitis C virus (HCV) antibody positivity, concomitant complications of cirrhosis, HCC including stages of Barcelona Clinic Liver Cancer (BCLC), hypertension, hemoglobin level, the amount of blood transfusion, cumulative dose of terlipressin, and the amount of free water hydration.

### 3.2. Changes in Serum Sodium Concentration

The overall change in serum sodium concentration and the mean lowest serum sodium concentration were 3.4 ± 9.6 mmol/L and 132.4 ± 8.8 mmol/L during treatment, respectively.

To determine the factors associated with the development of hyponatremia during terlipressin treatment, eighty-eight patients with hyponatremia were categorized into the three groups described above (Table 2).

Forty-seven patients (53.5%) exhibited mild decreases (between 5 and 10 mmol/L), 20 patients (22.7%) exhibited moderate decreases (between 10 and 15 mmol/L), and 21 patients (23.8%) exhibited severe decreases in serum sodium (greater than 15 mmol/L). The mean baseline serum sodium concentrations in the groups of mild, moderate, and severe decreases were 137.28, 136.96, and 139.9 mmol/L, respectively. The mean lowest serum sodium concentrations in the groups of mild, moderate, and severe decreases were 129.98, 124.25, and 118.57 mmol/L, respectively. The majority of patients who developed hyponatremia exhibited recoveries toward baseline serum sodium concentration after the end of treatment. The recovery time of the serum sodium concentration was 2.5 ± 1.8 days after the end of treatment. The patients who developed hyponatremia had higher baseline serum sodium levels than the patients in the other groups. There were inverse associations between the changes in the baseline serum bilirubin, MELD score, and Child-Pugh score and the changes in the serum sodium levels. The patient who developed severe hyponatremia exhibited the lower serum bilirubin levels, lower MELD scores, and lower Child-Pugh scores relative to the patients who developed mild hyponatremia, who had the highest serum bilirubin levels, the highest MELD scores, and the highest Child-Pugh scores. There were no significant differences between groups with respect to the previous histories of ascites and hepatic encephalopathy, baseline hemoglobin, baseline serum albumin, international normalized ratio, serum creatinine, blood transfusion amount, cumulative terlipressin dose, or the amount of free water hydration.

One patient developed mental changes with seizure coincidence with a marked reduction in serum sodium levels from 135 to 114 mmol/L within the first 48 hours of terlipressin treatment. This patient did not exhibit any abnormalities on brain computed tomography or electroencephalogram. The serum sodium levels and mental status of this patient did not improve despite the administration of 3% hypertonic saline. However, this patient successfully recovered the serum sodium concentration and mental status within 36 hours after terlipressin withdrawal.

### 3.3. Predictive Factors for the Development of Hyponatremia following Terlipressin Treatment

Univariate and multivariate logistic regression models were used to identify the independent risk factors that were significantly associated with development of hyponatremia during terlipressin treatment. In the univariate analysis, hepatitis B virus (HBV) positivity, DM, baseline serum creatinine level, baseline serum sodium level, baseline Child-Pugh score, and shock on admission were the candidate variables that were identified for the multivariate analysis (*P* < 0.05). Other clinical factors, such as age, gender, alcohol consumption, infectivity of HCV, ascites, hepatic encephalopathy, spontaneous bacterial peritonitis, HCC, hypertension, cumulative dose of terlipressin, baseline hemoglobin level, and the amount of free water hydration, were not significantly associated with the development of hyponatremia during terlipressin treatment. In the multivariate analysis, baseline normal or near-normal serum sodium and creatinine levels were independent positive predictors of the development of hyponatremia. The presence of HBV, DM, and shock on admission was independent negative predictors of the development of hyponatremia (*P* < 0.05). The strongest predictor of the development of hyponatremia following terlipressin treatment was the baseline serum creatinine level (odds ratio [OR], 0.22; 95% confidence interval [CI], 0.10–0.46; *P* < 0.001; Table 3).

### 3.4. In-Hospital Mortality

Overall, 69 of the 264 patients (26.1%) died during hospitalization. The in-hospital mortality rate was significantly lower in the group that developed hyponatremia than in the control group (6.8% versus 35.8%; *P* < 0.001). In the univariate analysis, HBV infection, alcohol, hepatic encephalopathy, HCC, baseline serum sodium level, serum bilirubin, serum albumin, international normalized ratio (INR), baseline serum creatinine level, baseline Child-Pugh score, baseline MELD score, shock on admission, the amount of blood transfusion, and the development of hyponatremia after terlipressin treatment were the candidate variables that were identified for the multivariate analysis (*P* < 0.05). Other clinical factors, such as age, gender, HCV infection, ascites, spontaneous bacterial peritonitis, hypertension, DM, hemoglobin level, and the cumulative dose of terlipressin, were not found to be significantly associated with in-hospital mortality. In the multivariate analysis, the development of hyponatremia after terlipressin treatment (OR, 0.19; 95% CI, 0.07–0.61; *P* = 0.005), HCC (OR, 8.39; 95% CI, 2.81–25.05; *P* < 0.001), and baseline serum bilirubin (OR, 1.08; 95% CI, 1.01–1.15; *P* < 0.001) was found to be independent predictors of in-hospital mortality (Table 4).

## 4. Discussion

Recent studies have demonstrated that terlipressin treatment for variceal bleeding results in the development of hyponatremia in 35–67% of cirrhotic patients [9, 10]. However, other randomized studies have reported that patients with acute variceal bleeding develop hyponatremia at rates of 0% and 6% [15–18]. This difference might have been influenced by differences in the definitions and methods for the identification of hyponatremia and selection bias. Because the present study was a matched case-control study, we were unable to precisely compare the rates of the occurrence of hyponatremia. However, we were able to evaluate the risk factors for the development of hyponatremia in cirrhotic patients with terlipressin treatment. The multivariate analysis of the development of hyponatremia indicated that the presence of HBV, DM, baseline serum creatinine levels, and shock on admission was independent negative predictors of the development of hyponatremia and that baseline normal or near-normal serum sodium was independent positive predictor of the development of hyponatremia. Previous studies have demonstrated that lower MELD scores [9] and higher baseline serum sodium levels [9, 10] are important predictors of hyponatremia. The present study revealed similar results in that higher baseline serum sodium levels and relatively preserved liver function were associated with the development of hyponatremia. Interestingly, our results strongly suggested that the baseline serum creatinine level is the most important predictor of reductions in sodium level (OR, 0.22; 95% CI, 0.10–0.46; *P* < 0.001). Terlipressin is an effective drug for variceal bleeding and hepatorenal syndrome in cirrhosis because it vasoconstricts the splanchnic circulation via the V1 receptors [19], but vasopressin also exerts activity on the renal V2 receptors present in the renal collecting ducts; therefore, it causes antidiuresis and natriuresis [20, 21]. It is well known that in patients with hepatorenal syndrome, terlipressin is able to increase serum sodium concentration toward an improvement in renal perfusion and water excretion. However, the administration of single dose of terlipressin to patients with cirrhosis is associated with an increase in aquaporin-2 excretion and a reduction in solute-free water clearance, and these findings are consistent with the significant activation of the renal V2 receptors and antidiuretic actions in patients with cirrhosis [22]. Therefore, the underlying renal function may be an important factor in the reduction in serum sodium because the renal V2 receptors are preoccupied by endogenous vasopressin when patients have poor underlying renal function [10]. The present study revealed that HBV positivity was negatively associated with the development of hyponatremia after terlipressin treatment (OR, 0.54; 95% CI, 0.29–0.99; *P* = 0.05). HBV infection can cause HBV-associated glomerulonephritis, which is the most common extrahepatic disorder of chronic HBV infection due to immune complex-mediated injuries [23, 24]. Recently, HBV infection was found to promote apoptotic damage in human renal tubular cells by triggering a pathway of Fas upregulation [25] and to potentially affect renal V2 receptors in renal tubular cells [25]. The present study also demonstrated that DM was negatively associated with the development of hyponatremia after terlipressin treatment (OR, 0.47; 95% CI, 0.23–0.93; *P* = 0.03). Diabetic renal injury, which is one of the most important chronic renal diseases in the world, may also exhibit protective effects against the development of hyponatremia for the same reason this effect is present in HBV infection. Shock on admission was negatively associated with the development of hyponatremia (OR, 0.19; 95% CI, 0.06–0.61; *P* = 0.005). Shock has been identified as a common cause of acute kidney injury [26]. Ultimately, the development of hyponatremia in cirrhotic patients with variceal bleeding following terlipressin administration was mainly associated with renal function and other factors that influence renal function, although previous studies have not revealed associations between renal function and the development of hyponatremia due to small numbers of patients and retrospective designs [9, 10].

In the present study, the development of hyponatremia following terlipressin treatment (OR, 0.19; 95% CI, 0.07–0.61; *P* = 0.005), HCC (OR, 8.39; 95% CI, 2.81–25.05; *P* < 0.001), and baseline serum bilirubin level (OR, 1.08; 95% CI, 1.01–1.15; *P* = 0.017) was associated with in-hospital mortality. As expected, liver function at admission was a risk factor for in-hospital mortality. Indeed, because other studies have not enrolled patients with HCC, these studies were unable to describe the role of HCC in mortality or the occurrence of hyponatremia. However, in clinical settings, because HCC with and without portal invasion is the main cause of variceal bleeding, the present study was able to provide data regarding terlipressin treatment in HCC patients with variceal bleeding. The patients who developed hyponatremia after terlipressin treatment tended to exhibit lower in-hospital mortality, which may have been associated with the relatively preserved liver and renal functions of these patients who have been described above.

One of the 21 patients with severe hyponatremia developed seizures. This patient exhibited rapid recovery of the serum sodium level and the neurologic abnormalities without sequelae after the withdrawal of terlipressin administration. However, the serum sodium levels and mental status of the patient did not improve before the withdrawal of terlipressin administration despite the administration of 3% hypertonic saline. There have been several reports of cases of hyponatremia with seizure that have rapidly recovered nearly normal sodium levels within a day of the withdrawal of terlipressin [7, 8, 12]. However, Solà et al. reported on a patient with severe hyponatremia and seizure who developed an osmotic demyelination syndrome after a very rapid recovery of the serum sodium level secondary to the withdrawal of terlipressin and treatment with hypertonic saline [9]. Therefore, if cirrhotic patients with relatively healthy hepatic and renal conditions exhibit variceal bleeding following terlipressin therapy with mental changes, physicians should be suspicious of the development of hyponatremia, although the development of hyponatremia following terlipressin treatment is rapidly reversible and is not associated with in-hospital mortality.

The present study has several limitations. First, enrolled patients were heterogeneous because we did not exclude patients with HCC. However, the presence or absence of HCC was not associated with the development of hyponatremia, and this finding might suggest that HCC was not related to the development of hyponatremia after terlipressin administration. Second, this was a retrospective matched case-control design study. Thus, future large-scale prospective studies are needed. Despite these limitations, to our knowledge, the present study is the first to report an association between baseline renal function and hyponatremia following terlipressin therapy in the patients with cirrhosis and other factors that influence renal function.

In conclusion, terlipressin treatment is commonly applied in cases of variceal bleeding but may often cause hyponatremia. The presence of HBV, DM, the baseline serum sodium level, shock on admission, and particularly baseline renal function may be predictive of the development of hyponatremia after terlipressin treatment. The role of renal dysfunction and baseline hyponatremia as potential protective factors against terlipressin-induced hyponatremia should be further developed. Although the development of hyponatremia after terlipressin treatment is rapidly reversible and is not associated with in-hospital mortality, physicians closely monitor electrolytes daily to prevent the possible neurological complications associated with severe hyponatremia when cirrhotic patients with relatively preserved liver function and renal conditions who exhibit variceal bleeding are treated with terlipressin.

## Figures and Tables

**Table 1:** Baseline characteristics of the study population.

Characteristics	Hyponatremia (*n* = 88)	Control (*n* = 176)	*P* value
Age (years)	56.3 ± 10.3	57.5 ± 11.3	0.421
Males, *n* (%)	73 (83)	146 (83)	1.000
Etiology of cirrhosis, *n* (%)			
Alcohol	55 (62.5)	97 (55.1)	0.252
Hepatitis B virus	27 (30.7)	83 (47.2)	0.011
Hepatitis C virus	6 (6.8)	6 (4.5)	0.437
Concomitant complications of cirrhosis, *n* (%)			
Ascites	66 (75)	123 (69.9)	0.385
Hepatic encephalopathy	18 (20.5)	46 (26.1)	0.310
Spontaneous bacterial peritonitis	3 (3.4)	15 (8.5)	0.120
Hepatocellular carcinoma, *n* (%)	26 (29.5)	51 (29)	0.924
BCLC stage A/B/C/D, *n* (%)	7/6/3/10 (27/23/12/38)	8/7/11/25 (16/14/22/48)	0.336
Diabetes mellitus, *n* (%)	17 (19.3)	(47.9)	0.029
Hypertension, *n* (%)	14 (15.9)	16 (9.1)	0.093
Hemoglobin, g/dL	9.5 ± 2.0	9.2 ± 8.2	0.822
Bilirubin, mg/dL	2.3 ± 1.9	5.6 ± 7.6	<0.001
Albumin, g/dL	3.0 ± 0.6	2.8 ± 0.6	0.006
INR	1.6 ± 0.5	2.0 ± 1.2	0.001
Creatinine, mg/dL	0.8 ± 0.4	1.6 ± 1.4	<0.001
Initial sodium, mEq	137.8 ± 4.5	134.1 ± 7.2	<0.001
Lowest sodium, mEq	125.9 ± 6.8	137.2 ± 7.8	<0.001
MELD score	14.0 ± 5.0	20.7 ± 10.2	<0.001
Child-Pugh score	8.3 ± 2.3	9.5 ± 2.7	0.001
Shock on admission, *n* (%)	4 (4.5)	45 (34.3)	<0.001
Blood transfusion (packed red cells)	3.2 ± 2.2	3.9 ± 3.6	0.118
Cumulative dose of terlipressin (mg)	17.9 ± 3.8	17.6 ± 7.5	0.621
Amount of free water hydration (L)	4.7 ± 2.3	4.05 ± 3.9	0.142

BCLC: Barcelona Clinic Liver Cancer; INR: international normalized ratio; MELD: Model for End-Stage Liver Disease; the data are expressed as the means ± the standard deviations (ranges) or numbers (%).

**Table 2:** Characteristics of the patients categorized according to changes in serum sodium concentration.

	Δ sodium 5–10 mEq (*n* = 47)	Δ sodium 10–15 mEq (*n* = 20)	Δ sodium > 15 mEq (*n* = 21)	*P* value
Ascites	32 (68)	19 (95)	15 (71)	0.460
Hepatic encephalopathy	7 (15)	7 (35)	4 (19)	0.462
Hemoglobin, g/dL	9.5 ± 1.9	9.7 ± 2.1	9.2 ± 2.3	0.766
Bilirubin, mg/dL	2.4 ± 2.1	2.9 ± 1.9	1.4 ± 0.7	0.024
Albumin, g/dL	3.0 ± 0.6	3.0 ± 0.5	3.1 ± 0.6	0.575
INR	1.6 ± 0.5	1.7 ± 0.6	1.5 ± 0.2	0.275
Creatinine, mg/dL	0.8 ± 0.4	0.9 ± 0.4	0.8 ± 0.3	0.632
Initial sodium, mEq/	137.3 ± 4.0	137.0 ± 4.5	140.0 ± 5.0	0.05
MELD score	14.0 ± 5.3	16.0 ± 5.9	12.2 ± 2.4	0.059
Child-Pugh score	8.3 ± 2.5	9.4 ± 2.1	7.5 ± 1.1	0.031
Blood transfusion (packed red cells)	3.3 ± 2.3	3.2 ± 2.3	3.1 ± 1.9	0.963
Cumulative terlipressin dose (mg)	18.0 ± 4.4	17.6 ± 3.6	18.0 ± 2.6	0.932
Amount of free water hydration (L)	4.3 ± 2.3	5.4 ± 2.6	5.0 ± 1.9	0.134

INR: international normalized ratio; MELD: Model for End-Stage Liver Disease; the data are expressed as the means ± the standard deviations (ranges) or numbers (%).

**Table 3:** Univariate and multivariate logistic analyses of the risk factors for the development of hyponatremia.

	Univariate analysis			Multivariate analysis		
	OR	95% CI	*P* value	OR	95% CI	*P* value
Hepatitis B virus positivity	0.50	0.29–0.85	0.011	0.54	0.29–0.99	0.05
Diabetes mellitus	0.50	0.27–0.93	0.027	0.47	0.23–0.93	0.03
Creatinine	0.17	0.08–0.35	<0.001	0.22	0.10–0.46	<0.001
Initial sodium	1.11	1.06–1.16	<0.001	1.09	1.03–1.17	0.005
Child-Pugh score	0.83	0.75–0.93	0.001	1.06	0.91–1.23	0.441
Shock on admission	0.14	0.05–0.40	<0.001	0.19	0.06–0.61	0.005

**Table 4:** Univariate and multivariate logistic analyses of the risk factors for the in-hospital mortality.

	Univariate analysis			Multivariate analysis		
	OR	95% CI	*P* value	OR	95% CI	*P* value
Hepatitis B virus positivity	2.27	1.30–3.97	0.031	0.38	0.14–1.08	0.069
Alcohol	0.50	0.29–0.87	0.014	0.45	0.19–1.08	0.075
Hepatocellular carcinoma	3.43	1.92–6.14	<0.001	8.39	2.81–25.05	<0.001
Hepatic encephalopathy	3.64	1.99–6.66	<0.001	1.61	0.69–3.78	0.273
Bilirubin	1.14	1.09–1.20	<0.001	1.08	1.01–1.15	0.017
Albumin	0.32	0.18–0.54	<0.001	0.95	0.42–2.17	0.907
INR	3.02	2.00–4.55	<0.001	1.64	0.94–2.89	0.08
Creatinine	1.83	1.37–2.44	<0.001	1.18	0.90–1.55	0.229
Initial sodium	0.90	0.86–0.94	<0.001	0.97	0.91–1.02	0.248
MELD score	1.15	1.11–1.20	<0.001			
Child-Pugh score	1.55	1.36–1.76	<0.001			
Development of hyponatremia	0.13	0.05–0.32	<0.001	0.19	0.07–0.61	0.005
Shock on admission	5.66	2.92–10.95	<0.001	1.06	0.94–1.20	0.369
Blood transfusion (PRCs)	1.10	1.01–1.19	0.028	1.06	0.94–1.19	0.369

INR: international normalized ratio; MELD: Model for End-Stage Liver Disease. PRCs: packed red cells.

## Abbreviations

HCC: Hepatocellular carcinoma
DM: Diabetes mellitus
MELD: Model for End-Stage Liver Disease
HCV: Hepatitis C virus
HBV: Hepatitis B virus
OR: Odds ratio
CI: Confidence interval
INR: International normalized ratio
PRC: Packed red cell.
